# Supplementary material for: Exploring ‘generative mechanisms’ of the antiretroviral adherence club intervention using the realist approach: a scoping review of research-based antiretroviral treatment adherence theories
Source: BMC Public Health. 2017 May 4;17:385. doi: 10.1186/s12889-017-4322-8 (PMC5418699; doi:10.1186/s12889-017-4322-8)
Supplement: Supplementary file 1 — Data extraction process. This table describes the characteristics and findings of the various studies included in the review. (DOCX 54 kb) [file 12889_2017_4322_MOESM1_ESM.docx]

**Additional File 1**: Data extraction table

| Study citation  Study Location | Study purpose and theory of interest | Study design and methods | Study limitations | Conclusions/Recommendations |
| --- | --- | --- | --- | --- |
| 1. Amico et al. (2005). An empirical test of the Information, Motivation and Behavioral Skills model of antiretroviral therapy adherence   Puerto Rico – North America | To evaluate the **Information-Motivation-Behaviour model**, which offers a dynamic, multivariate account of ART adherence. | - Cross-sectional survey - Used the Adherence to Combination Therapy Questionnaire to assess rates of adherence - Structural Equation model test - Sample: **200** HIV-positive patients on ART in 4 HIV clinics | - The study had a relatively small sample size - The study was also found within a single geographical locality and unclear whether it could be generalised to other populations, cultures and health systems such as in sub-Saharan Africa | Results of the current study provided strong support for the IMB model of ART adherence. Evaluation of a full, non-mediated model indicated that information and motivation were positively associated with adherence-related behavioural skills and that behavioural skills were in turn positively related to optimal adherence. The model also specifies that the relations between adherence-related information and motivation and adherence behaviour are mediated by adherence-related behavioural skills. The study offers support for the IMB model of adherence and furthers efforts to understand ART adherence as a dynamic behaviour that occurs in the context of an individual’s personal, social and environmental context. |
| 1. Amico et al. (2009). The Information–Motivation–Behavioral Skills Model of ART Adherence in a Deep South HIV+ Clinic Sample   USA – North America | To assess the determinants of adherence behaviour  postulated by the **Information–Motivation–Behavioural Skills model** of ART adherence in a sample  of HIV-positive patients in Mississippi | - Cross-sectional survey - A computer-delivered audio-supported survey based on LifeWindows ART Adherence Questionnaire and A modified version of the 3-day Adult AIDS Clinical Trials Group self-report measure - Structural Equation model. - **149** HIV-positive patients on ART in a large public infectious disease clinic conveniently sampled | - The study had a relatively small sample size - The study was also found within a single geographical locality and unclear whether it could be generalised to other populations, cultures and health systems such as in sub-Saharan Africa - The use of convenient sampling method of the sample provides little evidence for generalisation of the results. | Results of the current study provided strong support for the IMB model of ART adherence. Structural equation modelling indicated that ART-related information correlated with personal and social motivation, and the two sub-areas of motivation were not inter-correlated. Intervening to improve or support adherence will likely be most effective with theory-based interventions that offer a full compendium of intervention strategies targeting potential barriers within each of the information, motivation, and behavioural skills dimensions. |
| 1. De Bruin et al. (2005). Theory- and Evidence-Based Intervention to Improve Adherence to Antiretroviral Therapy Among HIV-Infected Patients in The Netherlands: A Pilot Study   Switzerland – Europe | To describe and pilot-test an intervention evidence-based intervention conceptualised on the **theory of planned behaviour and on self-regulation theories** – an evidence-based intervention to improve adherence of HIV-infected patients with antiretroviral medication. | - Within-subject comparison design - Twenty-six (**26**) treatment-experienced patients - Adherence - Medication Event Monitoring System (MEMS) caps for at least 5 months - Process tracing and interviews with patients and health care providers | - The study was a pilot study, thus had a relatively small sample size. This compromised the chances of generalisation - Only patients who were willing to change their behaviour may have agreed to participate in this pilot study - The effects of AIMS counselling on adherence in this pilot study have been short-term and derived from a homogeneous group of people. | The AIM strategy focused on improving patients’ self-management capacities. As a result of the AIM strategy, most non-adherent patients improved their understanding of the importance of adherence to their goals in future life, became motivated to change behaviour, redefined action plans, increased self-monitoring of behaviour, generated and implemented solutions for problems, and successfully changed behaviour. Both health care professionals and patients positively evaluated the intervention protocol and the electronic measurement of adherence. |
| 1. DiIorio et al. (2009). Adherence to Antiretroviral Medication Regimens: A Test of a Psychosocial Model   USA – North America | To test a psychosocial model of medication adherence based primarily on **Social Cognitive Theory** among people taking antiretroviral medications | - Randomised control trial - **236** participants were included in the analysis. - Factor analysis was used to develop the constructs for the model, and structural equation modelling. | - The study was also found within a single geographical locality and unclear whether it could be generalised to other populations, cultures and health systems such as in sub-Saharan Africa | In the final model, self-efficacy and depression demonstrated direct associations with adherence, whereas stigma, patient satisfaction and social support were indirectly related to adherence through their association with either self-efficacy or depression. The results suggest that adherence interventions that support self-efficacy and address depressive symptoms (in patients with depressive symptoms) may function to improve desirable levels of medication taking. |
| 1. Fisher et al (2006). Involving Behavioral Scientists, Health Care Providers, and HIV-Infected Patients as Collaborators in Theory-Based HIV Prevention and Antiretroviral Adherence Interventions   USA – North America | To propose a theory-based collaboration based on the **Information–Motivation– Behavioural model** involving health care providers, behavioural scientists and HIV-infected patients in the design of interventions to affect patient behaviour. | - Elicitation research - consisting of focus groups, questionnaires, and expert informant interviews and document review. | - Methodological approach and methods poorly illustrated. | A model for the collaborative design, implementation and evaluation of provider-initiated behaviour change interventions in the HIV domain is presented. |
| 1. Fisher et al (2006). An Information–Motivation–Behavioral Skills Model of Adherence to Antiretroviral Therapy   USA – North America | To provide a comprehensive review of relevant research concerning factors associated with HAART adherence framed in the  **Information–Motivation– Behavioural model** | - Systematic review - The MedLine and PsycINFO databases were searched from 1996 through April 2004 and supplemented with hand search of publications and abstracts - **15** HAART adherence promotion interventions | - Methodological approach and the data identification and selection methods poorly illustrated. | The study introduced an information–motivation–behavioural skills approach to conceptualising HAART adherence and reviewed the considerable correlational and experimental evidence for this approach. Based on the literature consulted, there is consistent and convergent empirical support for the IMB model’s assertions concerning the roles of adherence-related information, motivation and behavioural skills in understanding and promoting HAART adherence, as well as for the hypothesised interrelations among IMB model components. |
| 1. Fisher et al (2008). The Information-Motivation-Behavioral Skills Model of Antiretroviral Adherence and Its Applications   Not applicable | To review the core hypotheses of the **Information Motivation–Behavioural model** of HAART adherence | - Theory-based evidence from expert opinion | - No scientific process to ascertain rigour | Interventions based on the IMB model published to date suggest that the IMB model of HAART adherence may be useful in defining the core content for interventions deployed within diverse groups, including those struggling with substance abuse and those with limited literacy. According to the authors, this protocol could be incorporated into clinical practice as a valuable tool in working with patients individually. |
| 1. Gross et al (2013). Managed Problem Solving for Antiretroviral Therapy Adherence: A randomised trial   USA – North America | To determine whether an intervention derived from **Problem-Solving Theory**, Managed Problem Solving (MAPS), would improve antiretroviral outcomes. | - Randomised investigator-blind trial of MAPS - Managed Problem Solving consists of 4 in-person and 12 telephone-based meetings with a trained interventionist - Sample: 180 enrolled, 91 randomised to MAPS and 89 to usual care. - Intention-to-treat analyses | - Small sample | The investigators found that Managed Problem Solving is an effective antiretroviral adherence intervention in the first year with a new regimen. It was equally effective at improving adherence in treatment-experienced and naıve patients and did not lose effect over time. According to the authors, implementation of MAPS should be strongly considered where resources are available. |
| 1. Holtzman et al. (2015). Mapping Patient–Identified Barriers and Facilitators to Retention in HIV Care and Antiretroviral Therapy Adherence to Andersen's Behavioral Model   USA – North America | To identify, compare and contrast patient-reported barriers and facilitators to retention and adherence separately, and evaluate how these barriers and facilitators map to **Anderson’s Behavioural Model**. | - Using purposive sampling 51 HIV-infected adults from two university-affiliated clinics and one community-based were selected. - Data collected through interview technique - Data were analysed for themes using a grounded theory approach - Mapped themes and sub-themes to Anderson's model. | - The sample primarily consisted of heterosexual racial/ethnic minorities residing in an urban setting. Their unique challenges can play an important role in the types of barriers identified which affects population generalizability or transferability - Patients’ responses may have been influenced by social desirability bias | The findings may be useful in the design of future interventions to improve retention and adherence. By mapping barriers and facilitators to ABM, the authors identified the patient, clinic, and health system-level targets for intervention. |
| 1. Horvath et al. (2014). An Empirical Test of the Information-Motivation-Behavioral Skills Model of ART Adherence in a Sample of HIV-Positive Persons Primarily in Out-of-HIV-Care Settings   USA – North America | To determine if the associations observed between **Information–Motivation– Behavioural model** constructs and adherence behaviours persist according to the IMB model in the presence of depression and current drug use. | - A computer-delivered audio-supported survey based on LifeWindows ART Adherence Questionnaire and a modified version of the 3-day Adult AIDS Clinical Trials Group self-report measure - 312 participants responded to the questionnaire - Path models were used to assess the fit of a saturated versus fully mediated IMB model of adherence. | - The study was found within a single geographical locality and unclear whether it could be generalised to other populations, cultures and health systems such as in sub-Saharan Africa | Using the revised IMB scales, IMB constructs were associated with adherence as predicted by the theory in all but one model (i.e., the IMB model operated as predicted among non-drug users, and those with and without depression). Among drug users, information exerted a direct effect on adherence but was not significantly associated with behavioural skills. |
| 1. Johnson et al (2003). Theory-Guided, Empirically Supported Avenues for Intervention on HIV Medication Non-adherence: Findings from the Healthy Living Project   USA – North America | To clarify correlates of non-adherence to ART and to provide theory-guided, empirically supported direction based on **Social Action Theory**. | - Cross-sectional interview study using a computerised interview. - Sample: 2765 HIV-positive adults taking ART recruited from clinics, agencies, and via media ads in four U.S. cities from June 2000 to January 2002. - Outcome: Computer-assessed self-reported antiretroviral adherence | - Reliance on self-reported data, the cross-sectional design, and the use of a nonprobability sample. - It is unclear how those who volunteer for a research study may differ from other HIV-positive adults in adherence rates and correlates. | From the perspective of SAT, the results of the multivariate analysis suggest key correlates of adherence from the theoretical domains of environmental context and self-regulation capacities, but none from the domain of internal affective states. Results support the need for multi-focused interventions to improve medication adherence that addresses logistical barriers, substance use, attitudes and expectancies, as well as skills building and self-efficacy enhancement. |
| 1. Johnson et al (2006). A Social Problem-Solving Model of Adherence to HIV Medications   USA – North America | To test an explanatory model of HIV medication adherence using a **Social Problem-Solving** (SPS) framework | - Computer assisted interviewing - Structural equation modelling techniques were employed to test hypothesised relationships and to evaluate overall fit of the model to the data | - The use of cross-sectional data with a non-probability sample precludes causal inferences and limits generalisations that can be made from these findings. | Findings offer rationale and direction for SPS interventions to enhance adherence by improving psychological health. Such approaches, if effective, have the potential to influence positively the psychological well-being and adherence, thereby maximising clinical benefit from treatment, which is linked to lower mortality from AIDS. |
| 1. Johnson et al (2006). Positive Provider Interactions, Adherence Self-Efficacy, and Adherence to Antiretroviral Medications Among HIV-Infected Adults: A Mediation Model   USA – North America | To explore a model of medication adherence in which the relationship between positive provider interactions and adherence is mediated by adherence **self-efficacy**. | - Cross-sectional survey - Computerised self-administered and interviewer administered self-reported questionnaire where used - Sample: 3,818 HIV-positive individuals in four cities (San Francisco, Los Angeles, New York City, and Milwaukee) - Bivariate and univariate regression analyses were conducted | - Self-reported levels of adherence are suspected of being inflated because of recall, social desirability, and other biases. - A convenient sampling method was used to recruit the sample from multiple sources in each city | In this study, adherence self-efficacy mediated the relationship between positive provider interactions and medication adherence. This indicates that, statistically, the relationship of provider interactions and adherence can be explained by adherence self-efficacy, suggesting that self-efficacy may be the mechanism of the relationship between the other two variables. Results suggest implications for improving provider interactions in clinical care, and future directions for clarifying interrelationships among provider interactions, adherence self-efficacy, and medication adherence are supported. |
| 1. Kalichman et al. (2001). HIV Treatment Adherence in Women Living with HIV/AIDS: Research Based on the Information-Motivation-Behavioral Skills Model of Health Behavior   USA – North America | To compare HIV-positive women who were currently taking antiretroviral medications and had missed at least one dose of their drugs in the past week with women who were completely adherent to their medications in the past week on measures of the  **Information–Motivation– Behavioural model**. | - Participants completed confidential surveys and interviews based on the constructs of the IMB model - Sample: 112 women living with HIV/AIDS recruited from community-based service agencies and infectious disease clinics. - Structural Equation model test was used to analyse data. | - The use of convenient sampling method of the sample provides little evidence for generalisation of the results. - Self-reported levels of adherence are suspected of being inflated because of recall, social desirability, and other biases. | Results showed that women who had missed at least one dose of their HIV medications in the past week reported lower intentions (motivation) to remain adherent and lower adherence self-efficacy (skills). In addition, women who had missed a dose of medication in the past week were more likely to have ever used devices and strategies to remind them of doses but were no more likely to use such strategies. Interventions that enhance treatment adherence motivation and build adherence skills may help improve HIV treatment adherence in women receiving anti-HIV therapies. |
| 1. Kalichman et al. (2011). Brief Behavioral Self-Regulation Counseling for HIV Treatment Adherence Delivered by Cell Phone: An Initial Test of Concept Trial   USA – North America | To test a single office session followed by four biweekly cell phone counselling sessions grounded in **behavioural self-management model** of medication adherence. | - Two-arm randomised trial - Participants were baseline assessed and followed with biweekly unannounced pill counts and 4-month from baseline computerised interviews | - The sample size for this trial was small and participants were only followed for a short period. - The participants were also drawn from a convenience sample thus not representative of people receiving ART, thus the findings could not be generalised to all people receiving ART. | The intervention demonstrated increased adherence from an average that was suboptimal to levels needed to suppress viral replication. Participants who received the self-regulation counselling had an average of 90% adherence or greater throughout the follow-up period, including two months after counselling finished. |
| 1. Kennedy et al. (2004). Adherence to HIV Medications: Utility of the Theory of Self-Determination   USA – North America | To test adherence to antiviral therapy using the **Self-Determination Theory** (**SDT**). | - Cross-Sectional Design - Sample: 205 HIV+ patients - Data collected by adapted questionnaires assessing the various components of SDT - A four-step structural equation modelling was conducted | - Bias as well as social desirability in reporting adherence behaviour on the part of the patients - The selection of the sample was through convenient sampling methods. | The resulting model highlights the importance of self-determination in maintaining adherence to ART, lending support to the value of this model in predicting health behaviours, including adherence to ART. Perceived competence is identified as the strongest direct predictor of adherence, and is associated with patients’ sense of autonomy surrounding their medication adherence. The results of this study lend support to the relationship between autonomy support and autonomous motivation, and their relationship to adherence. |
| 1. Munro et al. (2007). A review of health behaviour theories: how useful are these for developing interventions to promote long-term medication adherence for TB and HIV/AIDS?   Not applicable | To review **Behaviour Change Theories** applicable to long-term treatment adherence. | - Integrative review - Databases electronic search, reference chase and consultations with experts. - Narrative analysis | - The search strategies were unable to capture all the available data on tests of health behaviour theories - This review examines only theories constructed by researchers and does not explore the health theories held by those receiving treatment. | Health behaviour theories may shed light on the processes underlying behaviour change. However, an explicit theoretical basis is not always necessary for a successful intervention and further examination is needed to determine whether theory-based interventions in health care are more effective than those without an explicit theoretical foundation. This review contributes to advancing this field by describing the commonly cited health behaviour theories, presenting the evidence and critique for each; discussing the applicability of these theories to adherence behaviour; and highlighting several recommendations for research and theory development. |
| 1. Norton et al. (2010). Information-Motivation-Behavioral Skills Barriers Associated with Intentional versus Unintentional ARV Non-adherence Behavior among HIV-Positive Patients in Clinical Care   USA – North America | To test the efficacy of an **Information–Motivation– Behavioural model**-based ARV adherence support program for HIV-positive patients in clinical care. | - Randomised controlled trial - Sample: 327 HIV-positive participants - Participants completed an assisted computer-administered self-interview - A series of one-way ANOVAs and Chi-square analyses examined differences between participants | - Patients’ self-report on the ARV adherence questionnaire and “taking a break” behaviour item may be subject to response bias. - Causal inferences between” taking a break” behaviour and behavioural skills barriers cannot be inferred due to the cross-sectional nature of the data. - The study relied on a single-item response to classifying patients as intentional or unintentional non-adherent | The current results point to the need for adherence-promotion programmes to include skills-building and self-efficacy enhancing intervention modules in order to address” taking a break” behaviour that could occur in about 10% of a clinic sample. Based on the present findings, patient-provider discussions should also include an exploration of how limitations in behavioural skills may be influencing HIV-positive patients’ conscious awareness of and decision to ”take a break” from ARV medications. |
| 1. Phillips (2011). Antiretroviral Therapy Adherence: Testing a Social Context Model Among Black Men Who Use Illicit Drugs   USA – North America | To develop a theoretical  model based on **Eco-social theory** to understand the effects of social context  factors that influence antiretroviral therapy adherence among a sample of HIV-infected Black men who use illicit drugs | - First a review of previous studies on Black men on ART who use illicit drugs - Sample: 160 Black men who use illicit drugs - Multiple regression techniques and path analysis were used to test the model | - The use of a convenience sample of HIV-infected Black men who use illicit drugs recruited from a homeless shelter and drug treatment facilities limits the generalisation power of the findings - Bias as well as social desirability in reporting adherence behaviour on the part of the patients | Tolerability of ART was observed to have a greater indirect effect on ART adherence than a direct effect. A positive state of mind and current illicit drug use indirectly affected ART adherence; however, significance was not achieved. Implications for the use of this theoretical model to guide research, clinical practice and policy as part of a human rights approach to HIV disease are articulated. |
| 1. Remien et al. (2006). Moving from Theory to Research to Practice Implementing an Effective Dyadic Intervention to Improve Antiretroviral Adherence for Clinic Patients   USA – North America | To describe the development, content, and testing of SMART Couples antiretroviral adherence intervention that is grounded in the **Social Action Theory**. | - Randomised controlled trial at 2 HIV/AIDS outpatient treatment centres - Adherence assessed using MEMS - Intention-to-treat analyses to identify difference in the groups | - Only a brief intervention, a 4-session couple focused adherence programme was offered. - Sample size was limited to a single geographical location | The authors suggest that providers routinely encourage their patients to bring a partner or family member to routine clinic appointments to help foster concrete support. Doing so enables the partner or family member to become an effective member of the patient’s team and allows the patient to feel more understood and supported by the important people in his or her life. It is also recommended that providers consider integrating a focus on problem-solving activities with the patient and his or her support partner rather than ‘‘prescribing’’ solutions. |
| 1. Reynolds (2003). The problem of the antiretroviral adherence: a self-regulatory model for intervention   Not applicable | To identify possible mechanisms by which adherence-enhancing interventions work drawing from the **Self-Regulatory Model**. | - Theory-based evidence from expert opinion | - Did not go through a scientific process | The proposed model is based on the self-regulatory theory that makes explicit the processes underlying ARV adherence behaviour. The model may be used to guide the development of a multi-component intervention. Maximising adherence and achieving the full potential of the antiretroviral therapies demand multi-dimensional initiatives that address complex behavioural and biomedical issues |
| 1. Saal & Kagee (2011). The applicability of the Theory of Planned Behaviour in predicting adherence to ART among a South African sample   South Africa – Africa | To determine the extent to which the **Theory of Planned Behaviour** (TPB) was applicable in predicting medication adherence among South Africans receiving antiretroviral therapy. | - Sample: 101 patients receiving ART by convenient sampling at a peri-urban public hospital. - Adapted questionnaire - Hierarchical regression analysis testing linear combination of TPB | - The study questionnaire took on average almost one hour to complete, introducing the possibility of respondent fatigue. - Sample was limited to a single hospital in a peri-urban region | Findings reveal that the linear combination of attitudes towards adherence perceived behavioural control and perceived group norms explained 12 percent of the variance in intentions to adhere to ART. We also found a non-significant relationship between intentions to adhere to treatment and self-reported adherence. The results call into question the extent to which TPB is helpful in understanding a health-promoting behaviour such as medication adherence among South Africans receiving ART |
| 1. Starace et al. (2006). Adherence to Antiretroviral Therapy: An Empirical Test of the Information–Motivation–Behavioral Skills Model   Campania, Italy - Europe | To evaluate the **Information–Motivation– Behavioural** skills model of ART adherence. | - Cross-sectional design - Sample: 100 HIV-positive patients in clinical care. - A series of structural equation modelling were conducted. | - Limitations of the current study include the cross-sectional design a longitudinal design could account for dynamic characteristics of adherence behaviour - A sample size of 100 is somewhat smaller than those typically used in structural equation modelling. | Consistent with the model, adherence-related information and motivation related significantly to adherence-related behavioural skills, and behavioural skills related significantly to self-reported optimal adherence. The effects of information and motivation on self-reported adherence were mediated by adherence-related behavioural skills. According to the IMB model, optimally adherence can best be brought about and supported by a careful assessment of the patient’s level of ART information, motivation toward taking ART. |
| 1. Torija et al, (2015). The Information and Motivation and Behavioral Skills Model of ART Adherence among HIV-Positive Adults in Mexico   Mexico – South America | To characterise the **Information–Motivation– Behavioural** core constructs and identify correlates of ART adherence in an HIV-positive clinic sample in Mexico. | - Sample: 109 conveniently sampled HIV-positive patients attending monthly visits at a local public hospital - LifeWindows IMB ART Adherence Questionnaire was used to collect data. - Data was analysed using factor analysis, cross-sectional analyses and bivariate analyses. | - Selection bias with regard to having mostly patients who routinely attend their monthly clinic appointments - There was the possibility of participants offering social desirable answers. - The population of this study does not represent the HIV/AIDS epidemiological panorama in Mexico. | As predicted by the model, only behavioural skills had a direct association with all measures of self-reported adherence, and motivation was associated with behavioural skills. The information did not demonstrate significant relations to either motivation or behavioural skills, nor did it directly associate with adherence. Self-reported adherence did not associate with CD4 counts, nor did any of the IMB model core constructs. |
| 1. Vissman et al (2011). Applying the theory of planned behaviour to explore HAART adherence among HIV-positive immigrant Latinos: Elicitation interview results   USA – North America | To explore the influences on intention to adhere to highly active antiretroviral therapy among immigrant Latinos living with HIV/AIDS in the south-eastern USA using the **Theory of Planned Behaviour**. | - Qualitative: Community-based participatory research - Sample: **25** immigrant Latinos - Individual in-depth elicitation interviews - Content analysis was applied to the data | - Participant selection was based on a convenience sample limiting the generalisation of the findings to all immigrant Latinos - Selection and response bias could be introduced when using the hospital staff to conduct the interviews | Before developing interventions to change adherence, the the degree to which attitude, subjective norms, or perceived behavioural control influence that adherence should be considered. Within a client-counselling framework, personalised facilitators of adherence could be identified and harnessed, and counsellors would build social support networks relying on influential referent groups and consider the clients’ healthcare experiences within the US and in their countries of origin. |
| 1. Ware et al. (2006). Examining Theoretic Models of Adherence for Validity in Resource-Limited Settings: A Heuristic Approach   Uganda & Nigeria - Africa | To explore the use of a heuristic schema for examining the validity of the **Information-Motivation-Behavioural** skills model of adherence in resource-limited settings. | - Qualitative exploratory - Illustrative validation exercise - 4 analytic questions applying aspects of the IMB skills model based on qualitative data from Uganda and Nigeria | - Use of secondary data from other qualitative studies | Meaningful research and intervention efforts to support adherence to antiretroviral medications in resource-limited settings require valid theoretic models. New models may be constructed for such settings, or existing models may be applied. Before applying existing models in new sociocultural contexts, they should be examined for validity and adapted as necessary for a good fit. The authors offered a set of practical conceptual tools for examining adherence models for cross-cultural validity. |
