# Supplementary material for: Exploring ‘generative mechanisms’ of the antiretroviral adherence club intervention using the realist approach: a scoping review of research-based antiretroviral treatment adherence theories
Source: BMC Public Health. 2017 May 4;17:385. doi: 10.1186/s12889-017-4322-8 (PMC5418699; doi:10.1186/s12889-017-4322-8)
Supplement: Supplementary file 2 — Data code manual. This is the coding manual that was used to indentify the various aspects of the context-mechanism-outcome heuristic tool. (DOCX 12 kb) [file 12889_2017_4322_MOESM2_ESM.docx]

| Category | | Definition | Coding Rules |
| --- | --- | --- | --- |
| Actors | | These are the individuals, groups, and institutions who play a role in the implementation and outcomes of an intervention | This was coded as the actions or actual practices of an individual, group or institution. |
| Context | | Context refers to salient conditions that are likely to enable or constrain the activation of programme mechanisms. | Components of both the physical and the social environment that favour or disfavour the expected outcomes |
| Mechanisms | | This refers to any underlying determinants or social behaviours generated in certain contexts | Any explanation or justification why a service or a resource was used by an actor to achieve an expected outcome, or considered as a constraint |
| Outcomes | Immediate outcome | Describes the immediate effect of the adherence club programme activities | Immediate outcome typically refers to changes in knowledge, skills or awareness, as these types of changes typically precede changes in behaviours or practices. |
|  | Intermediate outcome | Intermediate outcomes refer to behavioural changes that follow the immediate knowledge and awareness changes. | Codes here define a move from direct outcomes to intermediate outcomes, identified through the indirect impact of the activity and accountability of the programme. |
|  | Long-term outcome | Refer to change in the medium- and long-term, such as a patient’s health status, and impact on community and health system | The codes here represent the further indirect impact of the activity demonstrating the lesser accountability of the programme. |

Additional File 2: Data coding framework
